# Supplementary material for: Ageing-associated long non-coding RNA extends lifespan and reduces translation in non-dividing cells
Source: EMBO Rep. 2024 Oct 2;25(11):4921–49. doi: 10.1038/s44319-024-00265-9 (PMC11549352; doi:10.1038/s44319-024-00265-9)
Supplement: Supplementary file 1 — Appendix [file 44319_2024_265_MOESM1_ESM.pdf]

APPENDIX

Page 2: Appendix Table S1

Page 3: Appendix Figure S1

Appendix Table S1: Primers for RT-qPCR, RT-PCR and PCR (*Drosophila*)

**Transcript**	**Forward**	**Reverse**
aal1	CGCGTATTCCCTTTGGTGTT	GGGTCCTACATTCTTGAGGCT
ckb2	TCCTAAGCGCTCAATCCCTTG	CCATTGCATTCGAGGTCCTG
ctu1	GACTACATGTGAGCGTTGCG	CACTTCCCAAACCGAGACCA
act1	TCCTCATGCTATCATGCGTCTT	CCACGCTCCATGAGAATCTTC
ppb1	CACCTGTCACTGTATGCGG	GATCCACGTAATCTCCAAGGAA
rpl1901	GACTTGCTGCATCCGTCC	TAACCAAACCGTCCTTAATCAAC
rpl1902	CGTCTTGCTGCCTCTGTTT	GACCAAACCATCCTTGACCAAT
rpl1801	CTGGAAGCAACACTGTTCTTG	TTACGGCCCTCAGAACGA
rpl1802	AGCGCTTCTCTCGAGGGT	CACCACCAGCCTTCAAGATG
rps5	CTACGTCGAGTCTCACCCCG	ATGTCCTTAACTTCAACGCCC
rps502	GGCAGCCTCTATCATCCCTAAA	AGAGATATCCTTCACTTCAACACCT
rps27	GCAGTTTCTTCATGGATGTCAAGT	CCCTCCATAAGACGAGCCTT
aal1-Gene_specific_RT-reaction_primer		CATGGAATACACTATCACGACAACAT
ppb1-Gene_specific_RT-reaction_primer		TCAGTCATTCTACTCGCGTAA
aal1_Dm-cDNA-PCR	TGAAACATTGTCATCTCTTGC	GGAATACACTATCACGACAAC
act_ Dm-cDNA-PCR		
Control_Forward_ Dm-cDNA-PCR	CCGGAGTATAAATAGAGGCGC	
Control_Reverse1_ Dm-cDNA-PCR		CCGGAGTATAAATAGAGGCGC
Control_Reverse2_ Dm-cDNA-PCR		CGGTACCCGCCCGGGATCAG

Dm-cDNA-PCR: Drosophila_melanogaster-cDNA-PCR

Appendix Figure S1: Biotinylated antisense oligo probes used to pull-down *aal1* in ChIRP experiments.

> aal1_ChIRP-MS_Probes

#probes 1, 3 and 7 show homology ≥ 13bp to other transcripts and therefore not used
#Probes 2, 4, 5, 6, and 8 were used.

```
1 5'tgaaacattgtcatctcttgcttgtgaaattgttgttggcgctgctttttttaatattctg
                        3'acactttaacaacaaccgcg 5'(PROBE 1)

61  agaaattaattttgctctcaaaattgcttatttaatattggctatcataaataattttat

121 tatttatttattttattttttattttttttacatatttgttgtctgtatacccatgctca
                               (PROBE 2) acaacagacatatgggtacg

181 attgtgtcaaataccctaataggtgcgatttttgatgaacaattgaagactttgttttat

241 ttcccactatagaaaattaacacccggtagttttgaaggctttacttattttagcgactg
            tatcttttaattgtgggcca (PROBE 3)

301 tgtgctctaattaattacaagctcgattcgtgaaaacttccttttttgccacgcagacttt
                                (PROBE 4) aggaaaaacggtgcgtctga

361 taattccgaaatttacacagccgagaagctgcctacagtaaacgaagtaataataaatgg

421 gcattgctaaagaattttgatgtttttgagaagtcgcattctacaatttaaattttggta
                              actcttcagcgtaagatgtt (PROBE 5)

481 gaaaatatattacagaaaacagtaccaagtcagctaacttttaagccgtaacgcgtattc

541 cctttggtgtttgtggtctgctagtttcttaatccagcattcaaaaacaaggtaatttaa
         ccacaaacaccagacgatca (PROBE 6)

601 ggtctagaaacattatttgtgacttacagatccgacatttaaaatggcaaaacagtatct
                                    (PROBE 7) ttaccgttttgtcataga

661 cgactagttggttaaacaaattttagcctcaagaatgtaggacccttcttataattttgt
    gc

721 taaaggaaaaattcaaaaaacattcatgttgtcgtgatagtgtattccatgaataaaaat
                                 acagcactatcacataaggt (PROBE 8)

781 cac 3'
```
