# Supplementary material for: Ageing-associated long non-coding RNA extends lifespan and reduces translation in non-dividing cells
Source: EMBO Rep. 2024 Oct 2;25(11):4921–49. doi: 10.1038/s44319-024-00265-9 (PMC11549352; doi:10.1038/s44319-024-00265-9)
Supplement: Supplementary file 7 — Source data Fig. 1 [file 44319_2024_265_MOESM7_ESM.zip › 1B/ReadMe.docx]

**Figure 1B:** RNA fold-changes (log_2_) for *aal1* at the onset of stationary phase (Stat 0d) and after 4 and 11 days in stationary phase (Stat 4d and 11d) relative to proliferating cells, based on strand-specific RT-qPCR with gene-specific primers (∆∆Ct method). The *aal1* RNA levels are normalized to the lowly expressed coding gene *ppb1.* Bars indicate the mean with the standard errors (SE) of three independent repeats (Bio_Reps).

**Method Details:** RNA was extracted using the TRIzol reagent (Invitrogen) as per manufacturer’s recommendations. In-tube DNaseI (Turbo DNase, Invitrogen) digestion and subsequent reverse transcription (RT) was performed with 1 µg RNA. Random primed cDNA was prepared with SuperScript III reverse transcriptase (Invitrogen) as per standard protocols and all samples had an equivalent RT- reaction. Strand-specific RT with gene specific primers were used for *aal1* transcript quantification with 120 ng/μl Actinomycin D added additionally in the RT reaction. RT-qPCR was performed in a QuantStudio 6 Flex Real-Time PCR System (Applied Biosystems) with Fast SYBR Green Master mix (Applied Biosystems), 1/5 diluted cDNA template and 250 nM primers as per manufacturer’s recommendations. Samples were run in triplicates (replicate) along with non-template and RT- controls and relative starting quantity was estimated using the ΔΔCt^1^ method. The *aal1* transcript levels across samples were normalized to the *ppb1* expression levels; *ppb1* is the least variable gene under many perturbations including stationary-phase and is comparatively lowly expressed^2^. Melt curve analysis was performed following amplification to confirm the specificity of amplicons over primer dimers. All primer pairs were initially assessed in a standard curve for efficiencies, and primer pairs with efficiencies of 90-110% were used. All primers used are listed in Supplemental Table 1.

References

1. Livak, K. J. & Schmittgen, T. D. Analysis of relative gene expression data using real-time quantitative PCR and the 2(-Delta Delta C(T)) Method. *Methods* **25**, 402-408, doi:10.1006/meth.2001.1262 (2001).
2. Pancaldi, V., Schubert, F. & Bähler, J. Meta-analysis of genome regulation and expression variability across hundreds of environmental and genetic perturbations in fission yeast. *Mol Biosyst* **6**, 543-552, doi:10.1039/b913876p (2010).
