# Supplementary material for: Ageing-associated long non-coding RNA extends lifespan and reduces translation in non-dividing cells
Source: EMBO Rep. 2024 Oct 2;25(11):4921–49. doi: 10.1038/s44319-024-00265-9 (PMC11549352; doi:10.1038/s44319-024-00265-9)
Supplement: Supplementary file 7 — Source data Fig. 1 [file 44319_2024_265_MOESM7_ESM.zip › 1C/ReadMe.docx]

**Left and Right panels of Figure 1C**

Chronological lifespan assays for the above panels were performed as described^1^. To determine the CLS, strains driven by a *P41nmt1* promoter (*aal1-pOE*) and respective controls (evc) were grown with or without 15 µM thiamine in EMMG medium whereas deletion mutants (*aal1∆*) and controls (wt) were grown in YES medium, each strain as three independent biological replicates. Day 0 was defined as the day the cultures reached a stable maximal cell density. The percentages of viable cells were measured by serial dilution and plating in YES plates in duplicates for each dilution. Colonies were counted and the probable number CFUs per ml was calculated. The percentage viability was calculated relative to that of the CFUs at Day 0 (100% cell survival). CFU measurements were made daily for YES cultures and every 3-4 days for EMMG cultures until cultures reached 0.1–1% of the initial cell survival. Means of three independent biological replicates, with each culture measured twice at each time point is shown in the plots (error bars represent standard error).

**References**

1. Roux, A. E. *et al.* Pro-aging effects of glucose signaling through a G protein-coupled glucose receptor in fission yeast. *PLoS Genet* 5, e1000408,
