# Supplementary material for: Ageing-associated long non-coding RNA extends lifespan and reduces translation in non-dividing cells
Source: EMBO Rep. 2024 Oct 2;25(11):4921–49. doi: 10.1038/s44319-024-00265-9 (PMC11549352; doi:10.1038/s44319-024-00265-9)
Supplement: Supplementary file 7 — Source data Fig. 1 [file 44319_2024_265_MOESM7_ESM.zip › 1E/ReadMe.docx]

**Figure 1E:** *Left graph*: Cells deleted for *aal1* (*aal1∆*) show a slightly decreased lag period but similar growth rate to wild-type cells (wt). Cells were grown in a microbioreactor and mean growth curves were fitted with *grofit*^1^, with SD from six independent repeats shown as shades. *Right graphs*: Quantitation of growth rates and lag periods for experiment in the left graph. Growth rate and lag period calculations were done using *grofit*^1^ and statistical significance was determined with one-way ANOVA followed by Dunnett’s test (*multcomp*)^2^ to correct for multiple testing of the comparisons of the lag periods and growth rates of *aal1∆* against wt in R^3^.

**Details:** The assays were performed in a BioLector microbioreactor (m2p-labs) at 32˚C with 1000 rpm shaking and 85% humidity in 48-well microtiter plates (FlowerPlates). The starter cultures were grown to mid exponential phase, diluted in EMMG to achieve initial OD_600_ of ~0.05 and 1400 µl cultures were incubated in hexaplicates per treatment/genotype with well placements completely randomised to minimise any positional and border effects. Growth (biomass accumulation) was monitored in real time, measuring every 10 min until the cultures reached stationary phase. The growth data were normalised to time 0. Mean growth curves were fitted and growth rates and lag periods were calculated with *grofit*^1^.

**References**

1. Kahm, M., Hasenbrink, G., Lichtenberg-Frate, H., Ludwig, J. & Kschischo, M. grofit: Fitting Biological Growth Curves with R. J Stat Softw 33, 1-21 (2010).
2. Hothorn, T., Bretz, F. & Westfall, P. Simultaneous inference in general parametric models. *Biom J* **50**, 346-363, doi:10.1002/bimj.200810425 (2008).
3. R Core Team. R: A Language and Environment for Statistical Computing. R Foundation for Statistical Computing. Vienna, Austria (2020).
