# Supplementary material for: Ageing-associated long non-coding RNA extends lifespan and reduces translation in non-dividing cells
Source: EMBO Rep. 2024 Oct 2;25(11):4921–49. doi: 10.1038/s44319-024-00265-9 (PMC11549352; doi:10.1038/s44319-024-00265-9)
Supplement: Supplementary file 8 — Source data Fig. 2 [file 44319_2024_265_MOESM8_ESM.zip › 2B/ReadMe.docx]

**Images shown in Figure 2B**

Single-molecule RNA fluorescence *in situ* hybridization (smRNA-FISH) was performed with antisense probes as described^1^. Since the *aal1* RNA is not detectable during exponential growth and performing smRNA-FISH is technically challenging in aging stationary phase cells^2^, we used the strain overexpressing *aal1* in its native locus (*aal1-gOE*). Cells were grown in EMMG to mid-exponential phase and were fixed in 4% formaldehyde. The cell wall was partially digested using zymolyase. Cells were permeabilized in 70% ethanol, pre-blocked with bovine serum albumin and salmon sperm DNA and incubated overnight with custom Stellaris oligonucleotide probes (Biosearch Technologies) labelled with CAL Fluor Red 610. Cells were mounted in ProLong Gold antifade mount with DAPI (Molecular Probes), and imaged on a Leica TCS Sp8 confocal microscope, using a 63x/1.40 oil objective. Optical z sections were acquired (0.3 microns z-step size) for each scan to cover the entire depth of cells. The technical error in FISH-quant detection was estimated at 6%–7% by quantifying the *rpb2* mRNA foci with two sets of probes labelled with Quasar 670 (These images are also included). Images in the figure 2B show fluorescence micrographs of single-molecule FISH experiments of *aal1-gOE* cells and *aal1-pOE* cells (ectopic overexpression of *aal1* from a plasmid under the thiamine-repressible *P41nmt1* promoter). The *aal1* RNAs are labelled in green and DAPI-stained DNA is shown in blue. Scale bars: 5 μm.

**References**

1. Sun, X. M. *et al.* Size-Dependent Increase in RNA Polymerase II Initiation Rates Mediates Gene Expression Scaling with Cell Size. *Curr Biol* 30, 1217-1230 e1217, doi:10.1016/j.cub.2020.01.053 (2020).
2. Ellis, D. A. *et al.* R-loops and regulatory changes in chronologically ageing fission yeast cells drive non-random patterns of genome rearrangements. *PLoS Genet* 17, e1009784, doi:10.1371/journal.pgen.1009784 (2021).
