# Supplementary figures and images for: Ageing-associated long non-coding RNA extends lifespan and reduces translation in non-dividing cells
Source: EMBO Rep. 2024 Oct 2;25(11):4921–49. doi: 10.1038/s44319-024-00265-9 (PMC11549352; doi:10.1038/s44319-024-00265-9)

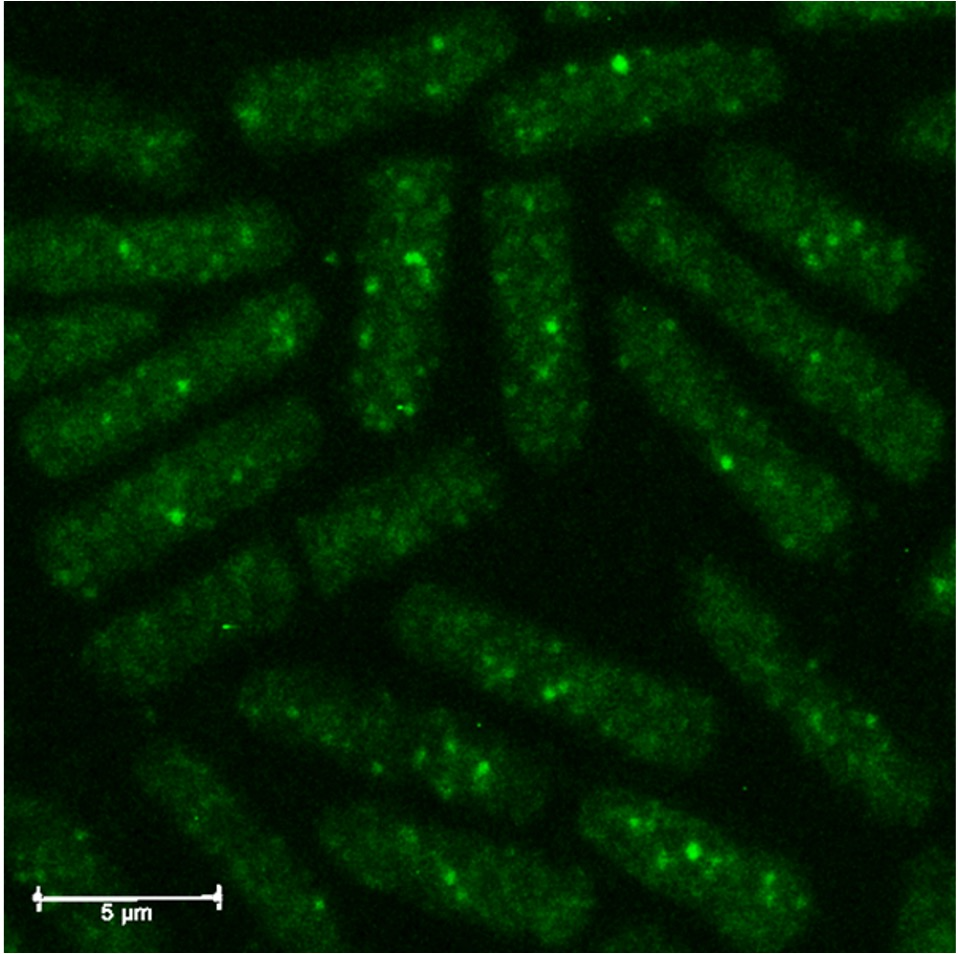

Supplement: Supplementary file 8 — Source data Fig. 2 [file 44319_2024_265_MOESM8_ESM.zip › 2B/aal1-gOE.tiff]

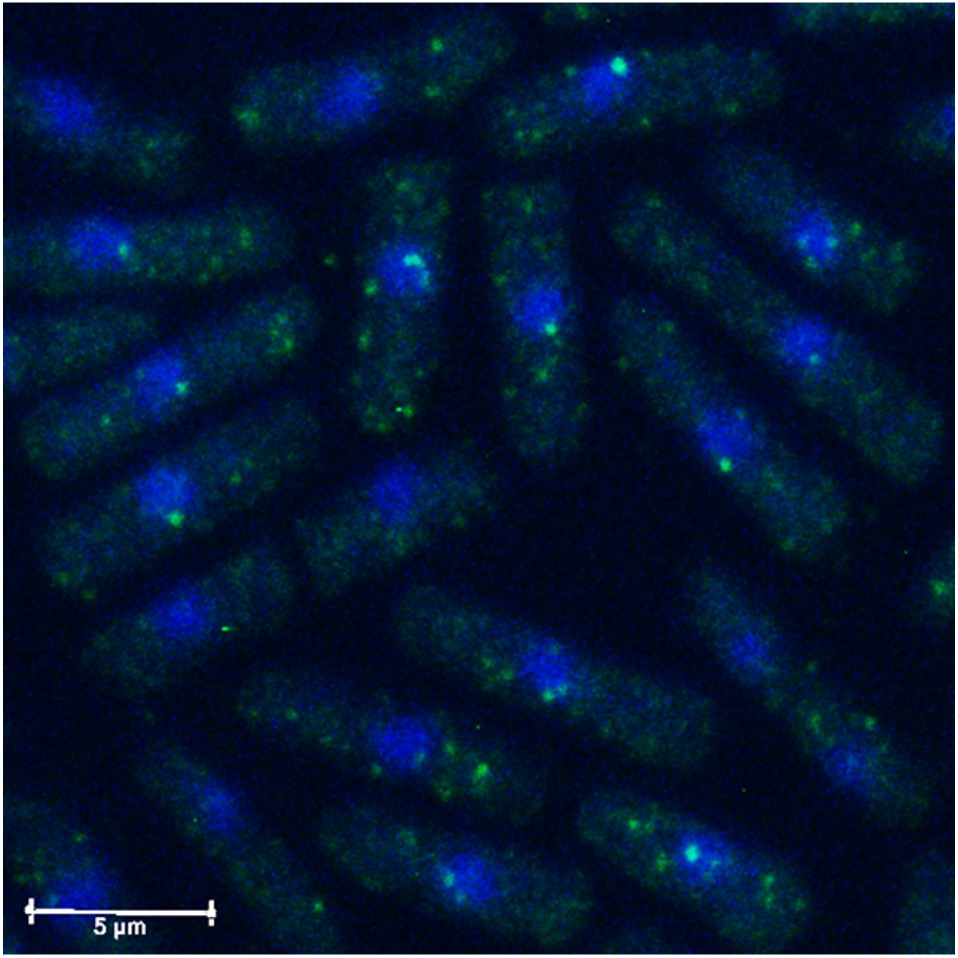

Supplement: Supplementary file 8 — Source data Fig. 2 [file 44319_2024_265_MOESM8_ESM.zip › 2B/aal1-gOE_with_DAPI.tiff]

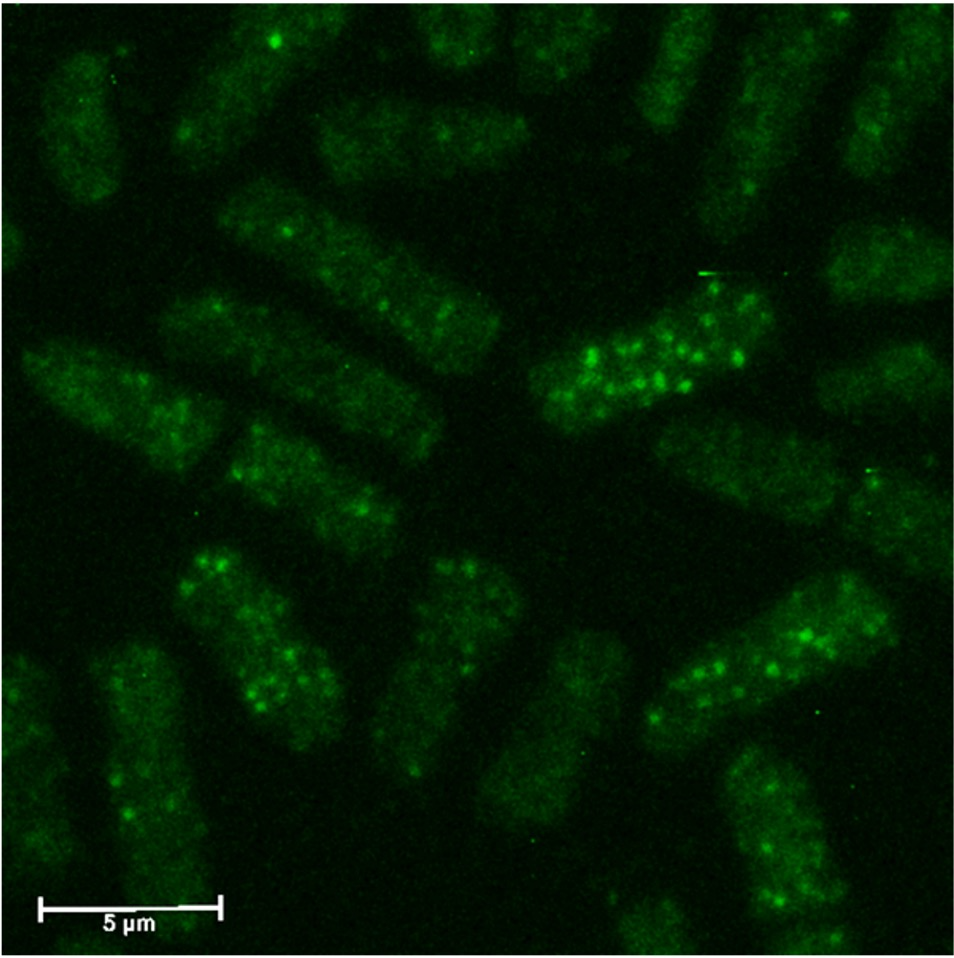

Supplement: Supplementary file 8 — Source data Fig. 2 [file 44319_2024_265_MOESM8_ESM.zip › 2B/aal1-pOE.tiff]

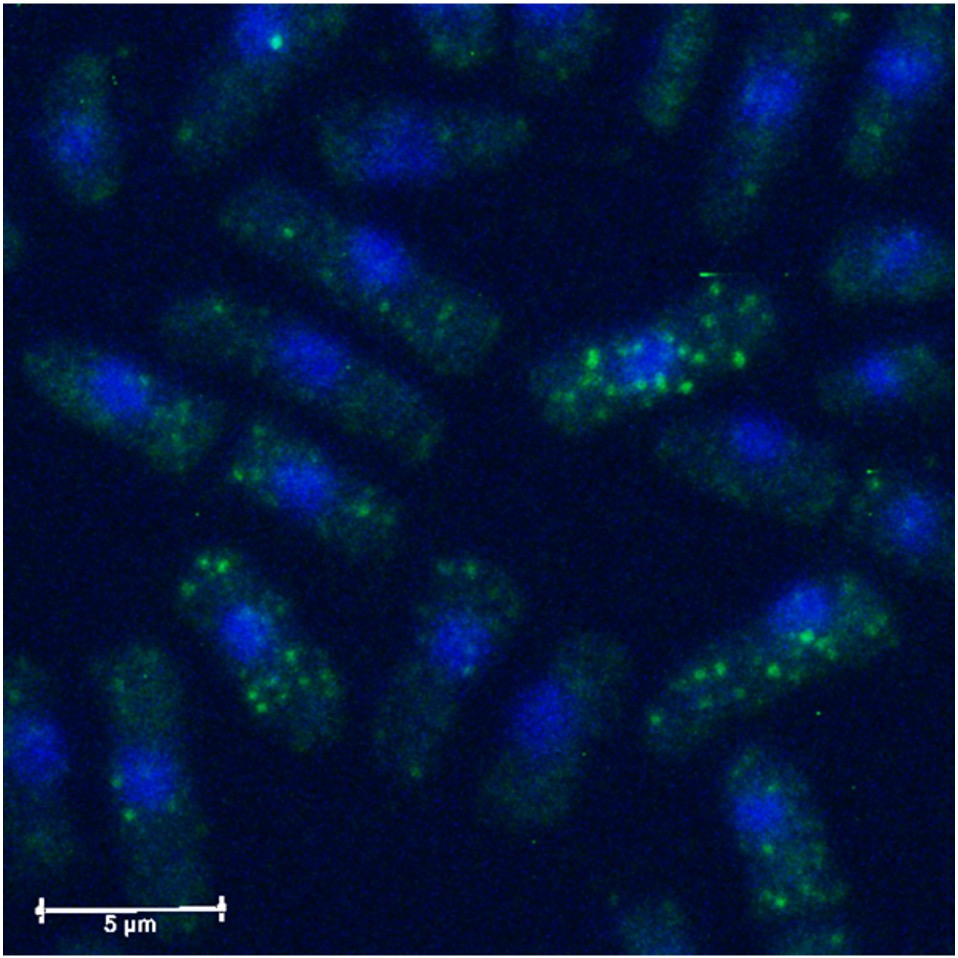

Supplement: Supplementary file 8 — Source data Fig. 2 [file 44319_2024_265_MOESM8_ESM.zip › 2B/aal1-pOE_with_DAPI.tiff]

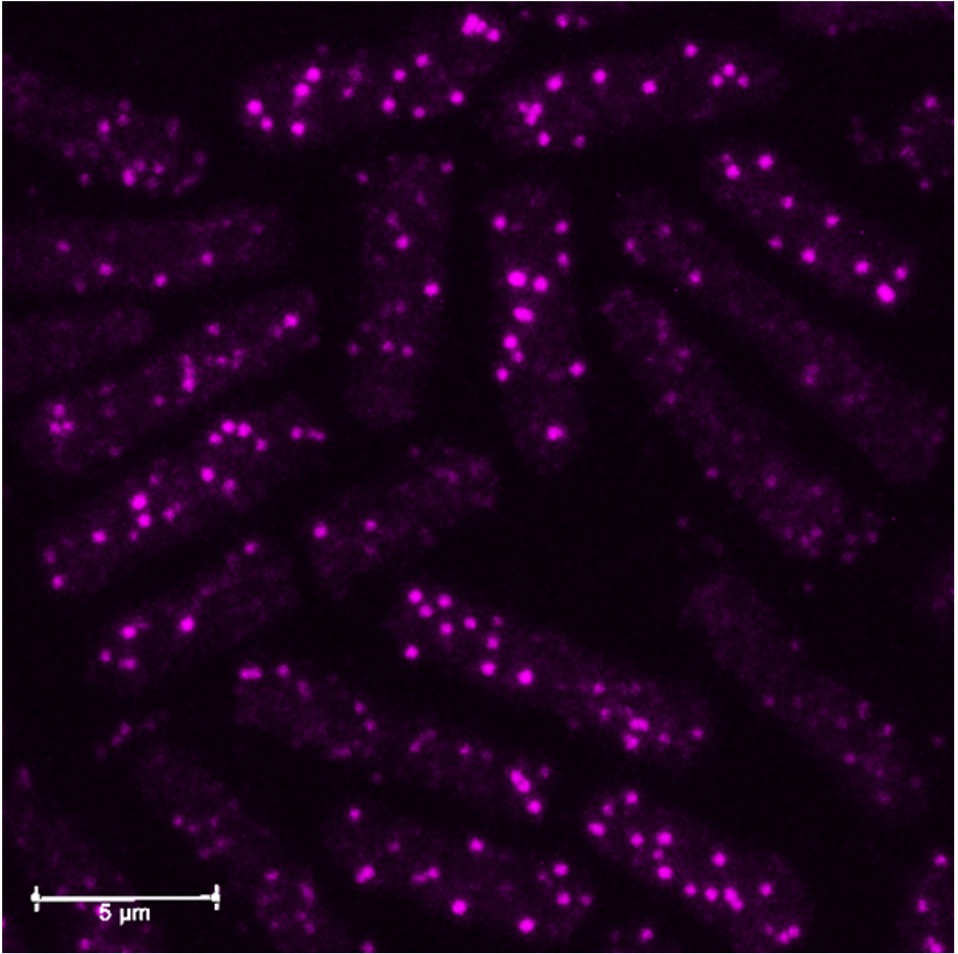

Supplement: Supplementary file 8 — Source data Fig. 2 [file 44319_2024_265_MOESM8_ESM.zip › 2B/rpb2_to_in_aal1-gOE.tiff]

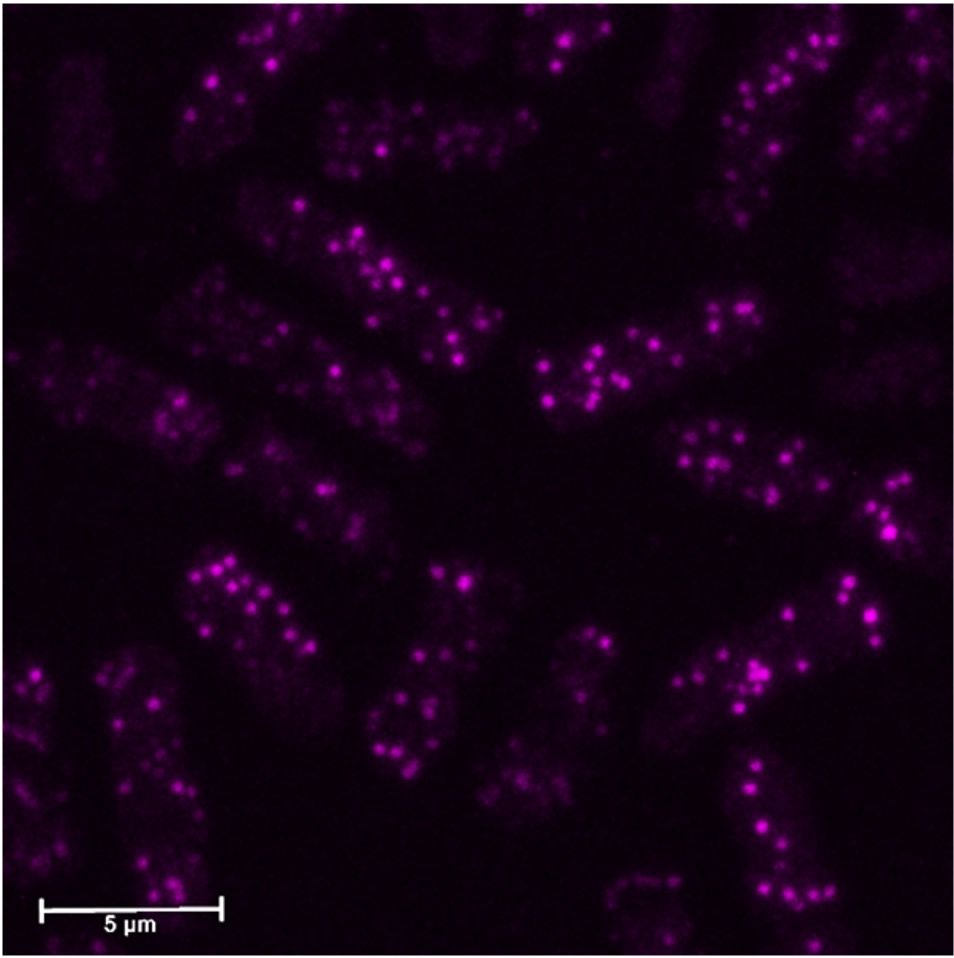

Supplement: Supplementary file 8 — Source data Fig. 2 [file 44319_2024_265_MOESM8_ESM.zip › 2B/rpb2_to_in_aal1-pOE.tiff]
