# Supplementary material for: Ageing-associated long non-coding RNA extends lifespan and reduces translation in non-dividing cells
Source: EMBO Rep. 2024 Oct 2;25(11):4921–49. doi: 10.1038/s44319-024-00265-9 (PMC11549352; doi:10.1038/s44319-024-00265-9)
Supplement: Supplementary file 9 — Source data Fig. 3 [file 44319_2024_265_MOESM9_ESM.zip › 3A/ReadMe.docx]

**Figure 3A:** The graph shows the proportions of genes with representative GO Biological Process terms among the 68 *aal1*-bound proteins compared to all *S. pombe* proteins (background). The associated Data file shows all GO terms for GO Biological Process, Cellular Component and Molecular Function (all terms for all three categories). GO terms were selected for non-redundancy, specificity, and significance, with the respective enrichment FDRs shown on top. FDRs were calculated with the Benjamini and Hochberg method in AnGeli^1^.

**Method Details**

The ChIRP-MS procedure which helped to identify the *aal1-*bound proteins is described in detail under methods. Differential protein enrichment analysis was performed with DEP^2^. As we observed that imputation of missing values with any available method in the package resulted in false positives, we adopted the following strategy. Any protein not identified in at least 2 out of 3 replicates of at least 1 strain was removed resulting in 218 proteins being identified. The remaining missing values were imputed with 1. All identified proteins were tabled in Dataset EV2. The data was background corrected and normalized by variance stabilizing transformation. A stringent cut-off of FDR ≤0.005 and log_2_ ≥6.5 in *aal1-pOE* and/or wild type relative to *aal1∆* was applied to eliminate *aal1*-RNA-independent background interactions. We identified 68 aal1-bound proteins at the end (Dataset EV2). The above 68 *aal1*-bound proteins are also more highly connected with each other than expected by chance based on known protein-protein interactions. Analysis of protein-protein interaction network with STRING^3^ for the 68 a*al1*-bound proteins reveals more connections than expected by chance (Figure 3B, *p=1.0e-16*). The following STRING parameters were used: active interaction sources = only experiments and databases; minimum required interaction score = high confidence (0.70); cluster with MCL; inflation=3. Dashed lines indicate lower confidence interactions.

**References**

1. Bitton, D. A. *et al.* AnGeLi: A Tool for the Analysis of Gene Lists from Fission Yeast. *Front Genet* 6, 330, doi:10.3389/fgene.2015.00330 (2015).
2. Zhang, X., Smits, A.H., van Tilburg, G.B., Ovaa, H., Huber, W. and Vermeulen, M. (2018) Proteome-wide identification of ubiquitin interactions using UbIA-MS. Nat Protoc, 13, 530-550.
3. Szklarczyk, D., Gable, A.L., Nastou, K.C., Lyon, D., Kirsch, R., Pyysalo, S., Doncheva, N.T., Legeay, M., Fang, T., Bork, P. et al. (2021) The STRING database in 2021: customizable protein-protein networks, and functional characterization of user-uploaded gene/measurement sets. Nucleic Acids Res, 49, D605-D612.
