# Supplementary material for: Ageing-associated long non-coding RNA extends lifespan and reduces translation in non-dividing cells
Source: EMBO Rep. 2024 Oct 2;25(11):4921–49. doi: 10.1038/s44319-024-00265-9 (PMC11549352; doi:10.1038/s44319-024-00265-9)
Supplement: Supplementary file 9 — Source data Fig. 3 [file 44319_2024_265_MOESM9_ESM.zip › 3C/ReadMe.docx]

**Figure 3C:** Polysome fractionation followed by RT-qPCR for *aal1-pOE* cells shows that *aal1* (red curve) mainly occurs with free ribosomal subunits (40S/60S) and monosomes (80S). The *ppb1* control mRNA (green curve) mainly occurs in polysomes. The corresponding polysome profile is shown as a black dashed line. Enrichment was calculated relative to the free RNA Fraction 2^1^. The plot shows the mean ± SE of three independent repeats of *aal1* overexpressing cells during exponential growth in minimal medium. The data shows the Ct values for all three independent replicates for aal1, ppb1 (mRNA control) and RT- (for aal1 primers) in separate sheets of the same .xlsx file. Data files also shows the percentage RNA enrichment calculations.

**Polysome fractionation followed by RT-qPCR details**

Cells were grown in EMMG at 32 ̊C. To block translation and capture translating ribosomes, cycloheximide (Sigma) was added to a final concentration of 100 µg/ml and incubated for 5 min with shaking. Cells were collected by centrifugation and lysed in lysis buffer (20 mM Tris-HCl pH 7.5, 50 mM KCl, 10 mM MgCl_2_) supplemented with 100 µM cycloheximide, 1 mM DTT (Sigma), 20 U/ml SuperaseIn (Invitrogen) and protease inhibitors (Complete, EDTA-free, Roche). Lysis was performed with 0.5 mm acid washed beads in a FastPrep instrument (MP, FastPrep24, Settings: speed, 6.0 m/sec; adapter, Quick Prep; time 20 sec; 5 cycles with ≥5 min incubations on ice in between). The lysates were centrifuged at 17,000 g for 5 min followed by another 15 min at 4˚C to remove cell debris, and the lysates were quantified in a Nanodrop (OD_260_). Equal amounts of each lysate were loaded for the polysome fractionation. Then, 10-50% linear sucrose gradients were prepared with a Gradient Master (Biocomp) using 10% and 50% sucrose (Sigma) solutions prepared in lysis buffer freshly supplemented with 100 µM cycloheximide and 1 mM DTT. The lysates were carefully laid on top of the gradients and centrifuged in a SW-41Ti rotor in a Beckman L-80 ultracentrifuge at 35K for 2 h 40 min at 4 ̊C. The tubes were processed in a Gradient Fractionator (Teldyne ISCO) with 55% sucrose as the chase solution. The polysome fractionation profiles were recorded and the fractions collected (~800-900 µl per fraction; 12-13 fractions per gradient) and immediately placed on ice.

RNA was extracted with TRIzol reagent (Invitrogen) as per manufacturer’s recommendations. RNA was precipitated with isopropanol overnight at -20 ˚C. DNase digestion and subsequent RT-qPCR analysis were performed with 1 µg RNA as described below. The calculation of *aal1* and *ppb1* enrichment across polysome profiles was calculated as described^1^. Briefly, the threshold cycle (C_T_) of each fraction was subtracted from the C_T_ of maximum value (always either fraction 1 or 2) for each primer set. The resulting difference in threshold cycles (∆C_T_) was used to calculate the relative change in mRNA levels between fractions by calculating the 2^∆CT^ value. The mRNA distribution across the entire polysome profile was graphically presented as the percentage of mRNA in each fraction divided by the total amount of mRNA (sum of 12 fractions).

References

1. Bachand, F., Lackner, D. H., Bähler, J. & Silver, P. A. Autoregulation of ribosome biosynthesis by a translational response in fission yeast. *Mol Cell Biol* **26**, 1731-1742, doi:10.1128/MCB.26.5.1731-1742.2006 (2006).
