# Supplementary material for: Ageing-associated long non-coding RNA extends lifespan and reduces translation in non-dividing cells
Source: EMBO Rep. 2024 Oct 2;25(11):4921–49. doi: 10.1038/s44319-024-00265-9 (PMC11549352; doi:10.1038/s44319-024-00265-9)
Supplement: Supplementary file 10 — Source data Fig. 4 [file 44319_2024_265_MOESM10_ESM.zip › 4F/ReadMe.docx]

**Figure 4F:** Polysome/monosome (P/M) ratios and relative ribosome content as in (4D) and (4E) for ectopic expression experiments shown in (4C). Relative ribosome content determined relative to wt+evc. Bars show mean ± SE of three independent repeats, with statistical significance determined using a two-sample t-test against the respective controls.

**Method Details**

Cells were grown in EMMG at 32 ̊C and 100 ml cultures were sampled for each timepoint. To block translation and capture translating ribosomes, cycloheximide (Sigma) was added to a final concentration of 100 µg/ml and incubated for 5 min with shaking. Cells were collected by centrifugation and lysed in lysis buffer (20 mM Tris-HCl pH 7.5, 50 mM KCl, 10 mM MgCl_2_) supplemented with 100 µM cycloheximide, 1 mM DTT (Sigma), 20 U/ml SuperaseIn (Invitrogen) and protease inhibitors (Complete, EDTA-free, Roche). Lysis was performed with 0.5 mm acid washed beads in a FastPrep instrument (MP, FastPrep24, Settings: speed, 6.0 m/sec; adapter, Quick Prep; time 20 sec; 5 cycles with ≥5 min incubations on ice in between). The lysates were centrifuged at 17,000 g for 5 min followed by another 15 min at 4˚C to remove cell debris, and the lysates were quantified in a Nanodrop (OD_260_). Equal amounts of each lysate were loaded for the polysome fractionation. Then, 10-50% linear sucrose gradients were prepared with a Gradient Master (Biocomp) using 10% and 50% sucrose (Sigma) solutions prepared in lysis buffer freshly supplemented with 100 µM cycloheximide and 1 mM DTT. The lysates were carefully laid on top of the gradients and centrifuged in a SW-41Ti rotor in a Beckman L-80 ultracentrifuge at 35K for 2 h 40 min at 4 ̊C. The tubes were processed in a Gradient Fractionator (Teldyne ISCO) with 55% sucrose as the chase solution. The polysome fractionation profiles were recorded. The area under the curve (AUC) of the monosome and the polysome peaks were measured with ImageJ^1^ for the calculation of the polysome:monosome ratios (P/M) and the total ribosome content (P+M). The P/M was calculated by dividing the sum of the AUC of all polysomes divided by the AUC of the monosome whereas P+M was the sum of both of these.

References

1. Schneider, C. A., Rasband, W. S. & Eliceiri, K. W. NIH Image to ImageJ: 25 years of image analysis. *Nat Methods* **9**, 671-675, doi:10.1038/nmeth.2089 (2012).
