# Supplementary material for: Ageing-associated long non-coding RNA extends lifespan and reduces translation in non-dividing cells
Source: EMBO Rep. 2024 Oct 2;25(11):4921–49. doi: 10.1038/s44319-024-00265-9 (PMC11549352; doi:10.1038/s44319-024-00265-9)
Supplement: Supplementary file 10 — Source data Fig. 4 [file 44319_2024_265_MOESM10_ESM.zip › 4E/ReadMe.docx]

**Figure 4E:** Relative ribosome content (Polysome+Monosome) in proliferating cells (Prolif), at the onset of stationary phase, Stat(0d), and after 6 days in stationary phase, Stat(6d), in *aal1-pOE*, relative to empty-vector control (evc) and *aal1∆* relative to wild type (wt) cells as indicated. Bars show mean ± SE of three independent repeats, with statistical significance determined using a two-sample t-test against the respective controls. The differences at Stat(6d) are not significant, likely reflecting that the ribosome content is generally low at this stage, which makes it harder to obtain reproducible measurements.

**Method Details**

Cells were grown in EMMG at 32 ̊C and 100 ml cultures were sampled for each timepoint. To block translation and capture translating ribosomes, cycloheximide (Sigma) was added to a final concentration of 100 µg/ml and incubated for 5 min with shaking. Cells were collected by centrifugation and lysed in lysis buffer (20 mM Tris-HCl pH 7.5, 50 mM KCl, 10 mM MgCl_2_) supplemented with 100 µM cycloheximide, 1 mM DTT (Sigma), 20 U/ml SuperaseIn (Invitrogen) and protease inhibitors (Complete, EDTA-free, Roche). Lysis was performed with 0.5 mm acid washed beads in a FastPrep instrument (MP, FastPrep24, Settings: speed, 6.0 m/sec; adapter, Quick Prep; time 20 sec; 5 cycles with ≥5 min incubations on ice in between). Number of lysis cycles were increased to ~12 for stationary phase and aging cells to achieve >80% lysis. The lysates were centrifuged at 17,000 g for 5 min followed by another 15 min at 4˚C to remove cell debris, and the lysates were quantified in a Nanodrop (OD_260_). Equal amounts of each lysate were loaded for the polysome fractionation. Then, 10-50% linear sucrose gradients were prepared with a Gradient Master (Biocomp) using 10% and 50% sucrose (Sigma) solutions prepared in lysis buffer freshly supplemented with 100 µM cycloheximide and 1 mM DTT. The lysates were carefully laid on top of the gradients and centrifuged in a SW-41Ti rotor in a Beckman L-80 ultracentrifuge at 35K for 2 h 40 min at 4 ̊C. The tubes were processed in a Gradient Fractionator (Teldyne ISCO) with 55% sucrose as the chase solution. The polysome fractionation profiles were recorded. The area under the curve (AUC) of the monosome and the polysome peaks were measured with ImageJ^1^ for the calculation of the total ribosome content (P+M). The P+M was calculated by adding the sum of the AUC of all polysomes and the AUC of the monosome.

References

1. Schneider, C. A., Rasband, W. S. & Eliceiri, K. W. NIH Image to ImageJ: 25 years of image analysis. *Nat Methods* **9**, 671-675, doi:10.1038/nmeth.2089 (2012).
