# Supplementary material for: Ageing-associated long non-coding RNA extends lifespan and reduces translation in non-dividing cells
Source: EMBO Rep. 2024 Oct 2;25(11):4921–49. doi: 10.1038/s44319-024-00265-9 (PMC11549352; doi:10.1038/s44319-024-00265-9)
Supplement: Supplementary file 10 — Source data Fig. 4 [file 44319_2024_265_MOESM10_ESM.zip › 4G/ReadMe.docx]

**Figure 4G:** Measurement of protein translation using puromycin labelling of proliferating cells. *Left*: graph showing anti-puromycin signal relative to anti-beta-actin signal quantified from chemiluminescence images for empty-vector control (evc) and *aal1-pOE* cells as well as wild type (wt) and *aal1∆* cells as indicated. The blot quantification details below. The quantified data were analysed with a linear regression model, which includes strain and batch as variables, and the significance was determined with a t-test. *Right:* Blots for signal quantification using anti-puromycin (left) and anti-beta-actin (right) antibodies for the 3 biological repeats of all strains as indicated above. Marker protein sizes in kilodaltons are indicated on the left.

**Method Details for Puromycin incorporation assay**

Assay was performed as described^1^. *S. pombe* cells were grown with constant shaking (180 rpm) at 32°C to mid-exponential phase in 25 ml EMM2 media. Puromycin (Gibco, #A11138-02) was added to a final concentration of 10 µM and incubated at 32°C for 30 min with shaking. Cells were harvested and snap-frozen in liquid nitrogen. Pellets were thawed on ice and broken with beads (Sigma, G8772) in protein lysis buffer (50 mM Tris, pH7.5, 150 mM NaCl, 5 mM EDTA, 10% glycerol, 1mM PMSF and protease inhibitor cocktail [cOmplete™ Mini EDTA-free, Roche]). Approximately 10 µg lysate was mixed with Laemmli buffer with freshly added 100 mM DTT and boiled at 95°C for 5 minutes. Proteins were separated on a gradient gel (Thermo Fisher Scientific, #NP0322BOX) and transferred onto a nitrocellulose membrane (Amersham) for 30 minutes using a semidry transfer system (Trans-Blot Turbo, BioRad). We used anti-puromycin antibody (Millipore, #12D10, 1:2500), HRP conjugated anti-mouse secondary (Abcam, #ab6789, 1:10,000) and anti-beta-actin antibody (ab8224, Abcam, 1:10,000). Blots were developed with Luminata Forte Western substrate (Millipore) and imaged in an Amersham ImageQuant800 imager. The intensity of anti-puromycin bands between 15 and 165 kDa was quantified from chemiluminescence images with the ImageQuant™ TL 10.2 analysis software with rolling ball background normalization. Expression was calculated relative to actin and compared to the respective controls for statistical significance. Quantification data for both Puromycin and beta-actin is provided in the Data file. The right panels show the whole blot with all three replicates for each shown genotype

References

1. Martinez-Miguel, V. E., Lujan, C., Espie-Caullet, T., Martinez-Martinez, D., Moore, S., Backes, C., Gonzalez, S., Galimov, E. R., Brown, A. E. X., Halic, M., Tomita, K., Rallis, C., von der Haar, T., Cabreiro, F. & Bjedov, I. Increased fidelity of protein synthesis extends lifespan. Cell Metab 33, 2288-2300.e12, doi:10.1016/j.cmet.2021.08.017 (2021).
