# Supplementary material for: Ageing-associated long non-coding RNA extends lifespan and reduces translation in non-dividing cells
Source: EMBO Rep. 2024 Oct 2;25(11):4921–49. doi: 10.1038/s44319-024-00265-9 (PMC11549352; doi:10.1038/s44319-024-00265-9)
Supplement: Supplementary file 11 — Source data Fig. 5 [file 44319_2024_265_MOESM11_ESM.zip › 5F/ReadMe.docx]

**Figure 5F:** Chronological lifespan assays for cells expressing *rpl1901* at different levels. *rpl1901* was expressed under the thiamine-repressible *P41nmt1* promoter at its native locus (*nmt1::rpl1901*), in the absence or presence of 3 or 15 µM thiamine as indicated. Only the moderate repression of *rpl1901* with 3 µM thiamine promotes lifespan extension. A Poisson distribution-based model was used for maximum likelihood estimates of the number of CFUs and shown in the y-axes as percentage relative to the CFUs at Day 0. Data points reflect the mean ± SE of three biological repeats.

**Method Details**

Cells were cultured in the minimal medium in the absence or presence of thiamine (3 µM or 15 µM), where the *P41nmt1* promoter is active or repressed, respectively. Day 0 was defined as the day the cultures reached a stable maximal cell density. The percentages of viable cells were measured using a robotics-based colony-forming units (CFU) assay^1^. A Poisson distribution-based model was used to obtain the maximum likelihood estimates for the number of CFUs, and percentage viability was calculated relative to that of the CFUs at Day 0 (100% cell survival). CFU measurements were made until cultures reached 0.1–1% of the initial cell survival.

References

1. Romila, C. A. *et al.* Barcode sequencing and a high-throughput assay for chronological lifespan uncover ageing-associated genes in fission yeast. *Microb Cell* **8**, 146-160, doi:10.15698/mic2021.07.754 (2021).
