# Supplementary material for: Ageing-associated long non-coding RNA extends lifespan and reduces translation in non-dividing cells
Source: EMBO Rep. 2024 Oct 2;25(11):4921–49. doi: 10.1038/s44319-024-00265-9 (PMC11549352; doi:10.1038/s44319-024-00265-9)
Supplement: Supplementary file 11 — Source data Fig. 5 [file 44319_2024_265_MOESM11_ESM.zip › 5E/ReadMe.docx]

**Figure 5E:** RT-qPCR experiment to quantify expression of *rpl1901* under the thiamine-repressible *P41nmt1* promoter at its native locus (*nmt1::rpl1901*), in the absence or presence of 3 or 15 µM thiamine as indicated. Expression was normalised to *act1* and shown relative to wild-type expression. Bars show the mean ± SE of three replicates.

**Method Details:** **RT-qPCR Analysis**

RNA was extracted using the TRIzol reagent (Invitrogen) as per manufacturer’s recommendations. In-tube DNaseI (Turbo DNase, Invitrogen) digestion and subsequent reverse transcription (RT) was performed with 1 µg RNA. Random primed cDNA was prepared with SuperScript III reverse transcriptase (Invitrogen) as per standard protocols. RT-qPCR was performed in a QuantStudio 6 Flex Real-Time PCR System (Applied Biosystems) with Fast SYBR Green Master mix (Applied Biosystems), 1/5 diluted cDNA template and 250 nM primers as per manufacturer’s recommendations. Samples were run in triplicates and relative starting quantity was estimated using the ΔΔCt[^121^](#_ENREF_121) method. *rpl1901* transcript levels were normalised to *act1* expression levels. Melt curve analysis was performed following amplification to confirm the specificity of amplicons over primer dimers. All primer pairs were initially assessed in a standard curve for efficiencies, and primer pairs with efficiencies of 90-110% were used for RT-qPCR. All primers used are listed in Supplemental Table 1.

References

1. Livak, K. J. & Schmittgen, T. D. Analysis of relative gene expression data using real-time quantitative PCR and the 2(-Delta Delta C(T)) Method. *Methods* **25**, 402-408, doi:10.1006/meth.2001.1262 (2001).
