# Supplementary material for: Ageing-associated long non-coding RNA extends lifespan and reduces translation in non-dividing cells
Source: EMBO Rep. 2024 Oct 2;25(11):4921–49. doi: 10.1038/s44319-024-00265-9 (PMC11549352; doi:10.1038/s44319-024-00265-9)
Supplement: Supplementary file 12 — Source data Fig. 6 [file 44319_2024_265_MOESM12_ESM.zip › 6A-B/ReadMe.docx]

**Figure 6A & 6B :** **(A)** Lifespans of female *Drosophila* with *UAS-aal1* induced in adult midguts with the gut-specific *TIGS* driver by RU486 feeding to activate expression (−RU486: n = 142 dead/8 censored flies, +RU486: n = 144 dead/6 censored flies, p = 0.003, log-rank test).

(**B**) Lifespans of control females carrying the *TIGS* driver alone with or without RU486 feeding (−RU486: n = 131 dead/19 censored flies, +RU486: n = 144 dead/6 censored flies, p = 0.3, log-rank test).

**Method Details**

We used an outbred wild-type stock that was initially collected from Dahomey (present Benin) in 1970 and subsequently maintained in large population cages on a 12hr:12hr light/dark cycle at 25˚C to maintain lifespan and fecundity at levels similar to wild-caught flies. The *white^1118^* mutation was introduced into this background to allow easier tracking of transgenes and Wolbachia infection was cleared by tetracycline treatment. Before the experiments, the *aal1* fly lines and gene-switch drivers (TIGS) were backcrossed into this white Dahomey (wDah) background for at least six generations. All stocks were maintained, and experiments were conducted at 25˚C and 60% humidity with 12hr:12 hr light/dark cycles, on SYA food containing 10% brewer’s yeast, 5% sugar, and 1.5 % agar with nipagin and propionic acid added as preservatives.

For lifespan assays, experimental flies were generated from suitable crosses in cages containing grape juice, agar, and live yeast. Flies were allowed to mate and the eggs were collected after 22 hrs and 20 µL of egg sediments (in 1xPBS) were seeded on SYA medium in glass bottles to rear flies at standardized larval densities. Flies emerged after 10 days and were transferred to new bottles where they were allowed to mate for 48 hrs before sorting females into experimental vials at a density of 15 flies per vial. To induce transgene expression using the GAL4/UAS GeneSwitch system, RU486 (Sigma, dissolved in ethanol) was added to the media at a final concentration of 200 µM. As a control (RU-), equivalent volumes of the vehicle alone were added. Flies were transferred to fresh vials three times a week and their survival scored (Figure 5A). To control for potential RU486 artifacts, driver-only controls feeding RU486 were included in the experiment (Figure 5B).
