# Supplementary material for: Ageing-associated long non-coding RNA extends lifespan and reduces translation in non-dividing cells
Source: EMBO Rep. 2024 Oct 2;25(11):4921–49. doi: 10.1038/s44319-024-00265-9 (PMC11549352; doi:10.1038/s44319-024-00265-9)
Supplement: Supplementary file 13 — Expanded View Figures [file 44319_2024_265_MOESM13_ESM.pdf]

Expanded View Figures

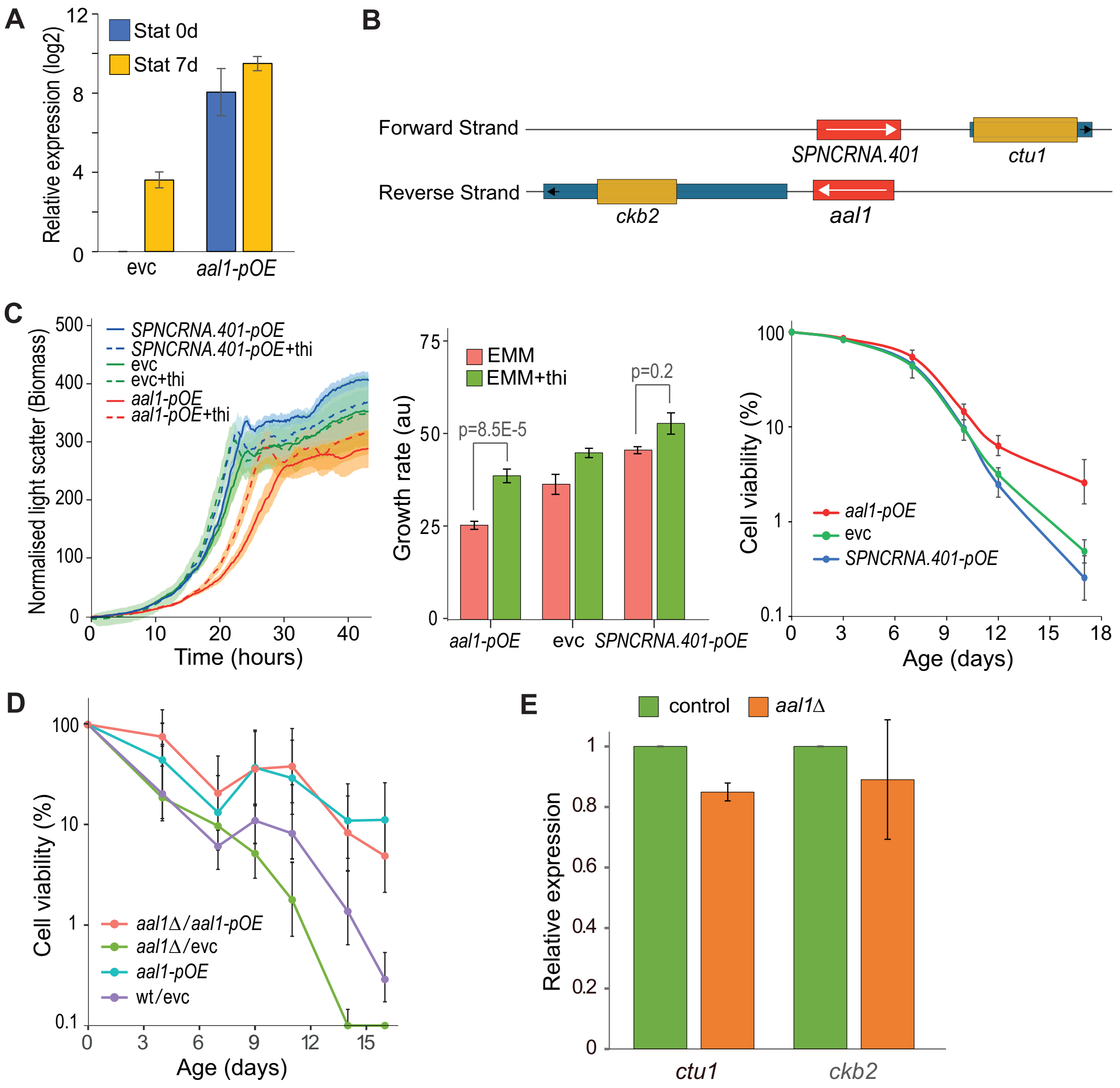

Figure EV1. Analysis of the *SPNCRNA.401* lncRNA.

(**A**) Expression of *aal1* in *aal1-pOE* cells at the onset of stationary phase (Stat 0d) and after 7 days in stationary phase (Stat 7d) relative to empty-vector control cells (evc) at the onset of stationary phase (Stat 0d), measured with strand-specific RT-qPCR. The *aal1* RNA levels are normalized to the lowly expressed coding gene *ppb1*. Bars indicate the mean±SD (standard deviation) of three independent repeats. (**B**) Genomic environment of *aal1* gene showing a 745 nucleotide overlap in antisense direction with the *SPNCRNA.401* gene. *Red boxes*: lncRNA genes, with the transcriptional direction indicated by white arrows; *ochre and blue boxes*: open reading frames and untranslated regions, respectively, of coding genes. Visualization using the PomBase genome browser (Harris et al, 2022). (**C**) Left graph: Growth assay for cells ectopically overexpressing *aal1* and *SPNCRNA.401* under the thiamine-repressible *P41nmt1* promoter (*aal1-pOE* and *SPNCRNA.401-pOE*) compared to empty-vector control (evc) with/or without 15 μM thiamine (thi) added to the medium. Experimental setup and analysis as in Fig. 1D. Middle graph: Quantitation of growth rate for experiments shown in the left graph, as in Fig. 1D. Right graph: CLS assays for *aal1-pOE* and *SPNCRNA.401-pOE* cells compared to empty-vector control (evc) cells. Experimental setup and analysis as in Fig. 1C. (**D**) CLS assays for *aal1Δ* cells ectopically overexpressing *aal1* (*aal1Δ/aal1-pOE*) compared to *aal1Δ* and wild-type cells overexpressing empty-vector controls (*aal1Δ*/evc; wt/evc) and *aal1-pOE* cells. (**E**) Expression of genes flanking *aal1* in the presence and absence of *aal1*. RT-qPCR experiment to determine transcript levels of *ctu1* and *ckb2* in *aal1Δ* cells relative to wild-type cells (control). Four independent biological repeats were carried out using 7-day-old stationary-phase cells. Data were normalized to *act1* expression. Expression of *ctu1* was slightly lower in *aal1Δ* compared to control cells ($p_{Student's\ T} \sim 0.001$), while expression of *ckb2* showed no significant difference ($p_{Student's\ T} \sim 0.35$).

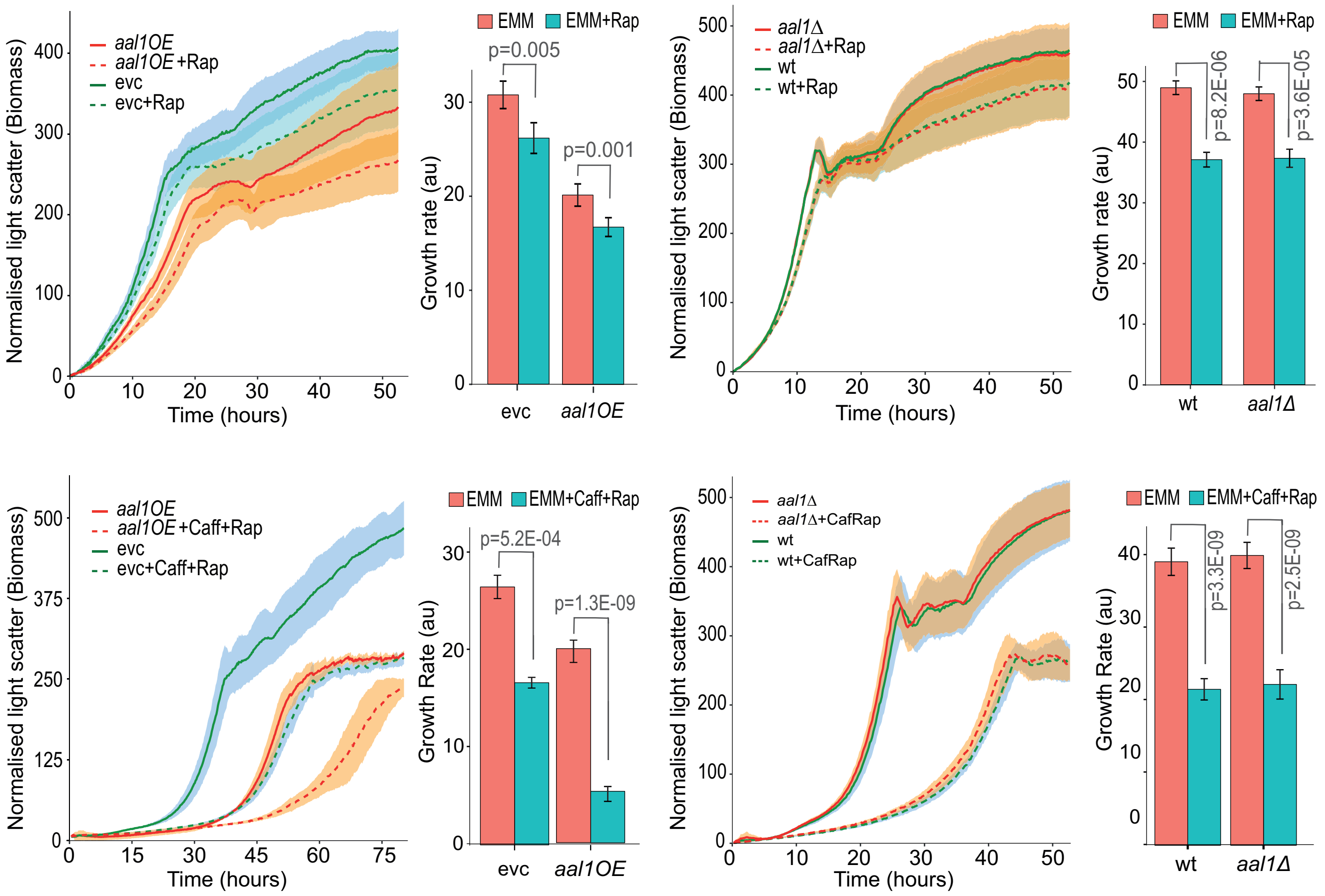

Figure EV2. ***aal1* phenotypes do not depend on TORC1 signalling.**

Top graphs: Rapamycin inhibits cell growth in *aal1-pOE* and *aal1Δ* mutants to a similar degree as in the respective controls, indicating that TORC1 and *aal1* functions exert additive effects. Rapamycin (300 ng/ml) was added after 8 h of initial growth to avoid overly long lag periods. Bottom graphs: The combination of caffeine (10 mM) and rapamycin (100 ng/ml) leads to a stronger inhibition of cell growth in *aal1-pOE* and *aal1Δ* mutants, similar as in the respective controls, indicating again that TORC1 and *aal1* functions have additive effects. Cells were grown in a microbioreactor and mean growth curves were fitted with *grofit* (Kahm et al, 2010), with SD shown as shades. Quantitation of growth rates (mean ± SE) for experiments is shown in the bar graphs. Details as in Fig. 1D,E.

 © The Author(s)

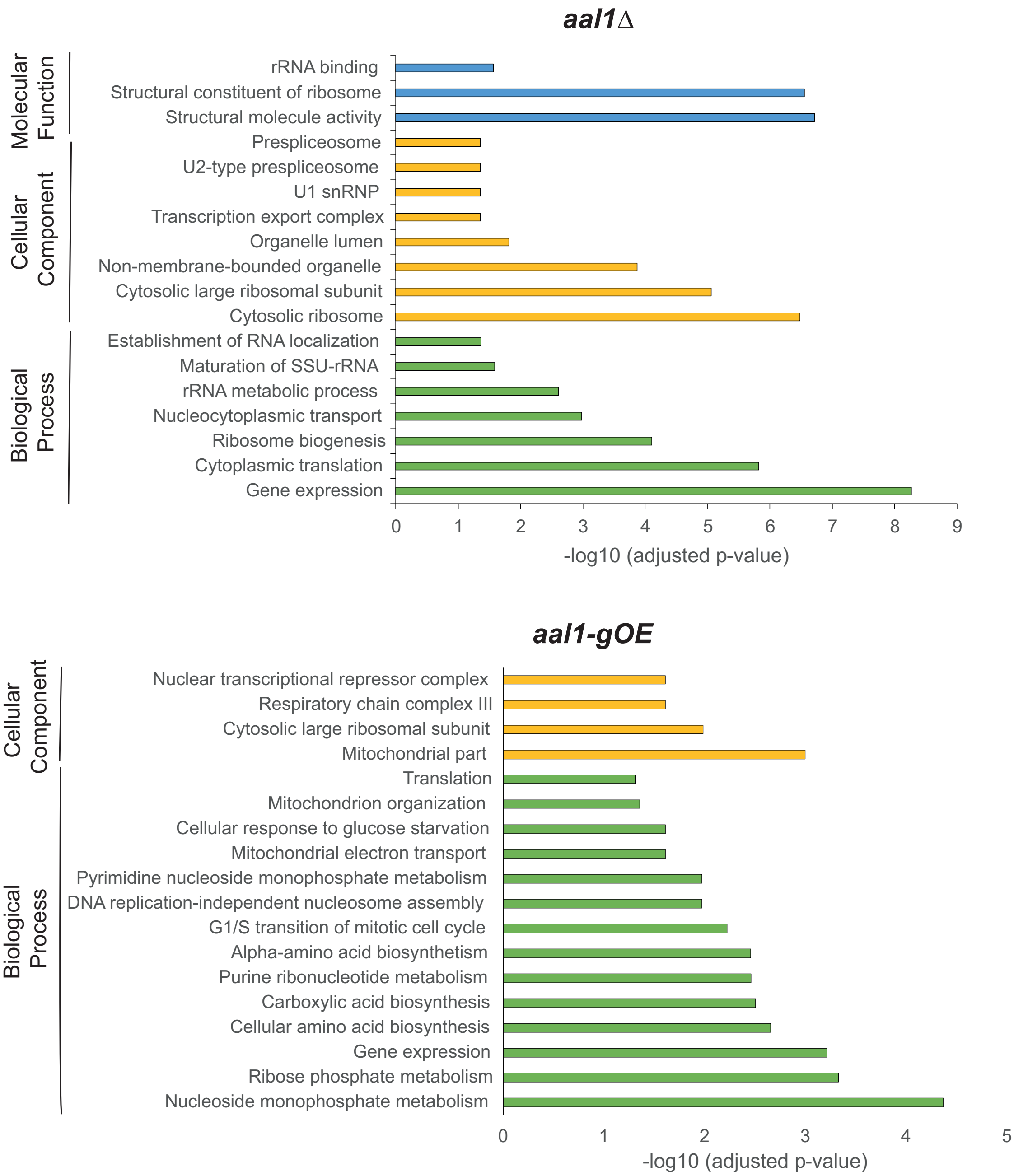

Figure EV3. Functional enrichments among genes genetically interacting with *aal1Δ* and *aal1-gOE* mutants.

GO-term enriched among the genes that showed positive or negative genetic interactions (adjusted *p*-value[FDR] ≤0.05) in at least 2 of the 3 repeats in the SGA screens using *aal1Δ* (top) or *aal1-gOE* (bottom) as query mutants (see Methods). Representative GO terms for Biological Process, Molecular Function, and Cellular Component are shown, selected for non-redundancy, specificity, and significance. The graphs show the $-\log_{10}$ of adjusted *p*-values (false-discovery rate) for enrichment of the different terms. Visualisation with ShinyGO (ver 0.77). The genetic-interaction and background gene lists are provided in Dataset EV1.

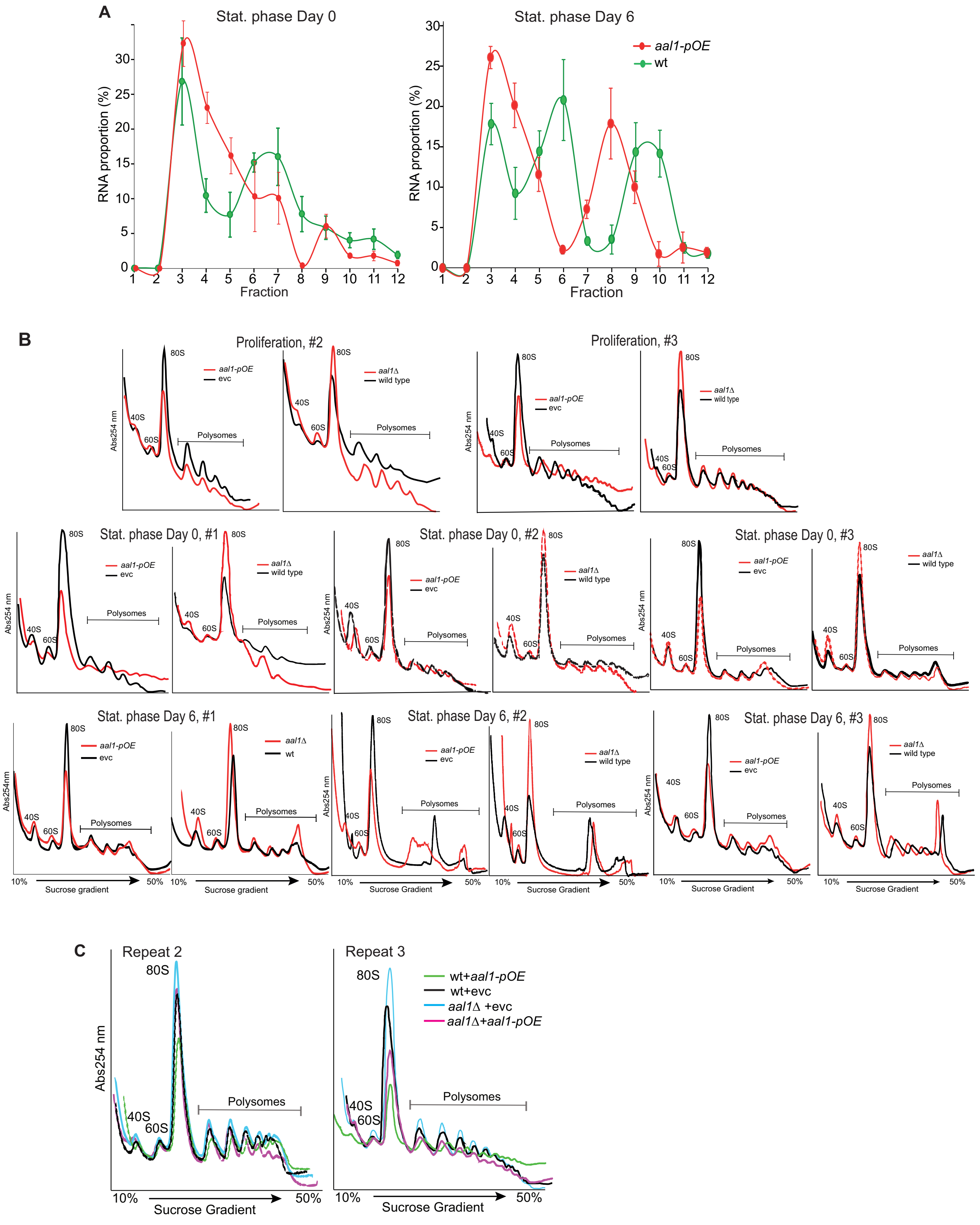
A
Stat. phase Day 0
Stat. phase Day 6
RNA proportion (%)
Fraction
aal1-pOE
wt
B
Proliferation, #2
Proliferation, #3
Stat. phase Day 0, #1
Stat. phase Day 0, #2
Stat. phase Day 0, #3
Stat. phase Day 6, #1
Stat. phase Day 6, #2
Stat. phase Day 6, #3
aal1-pOE
evc
aal1Δ
wild type
wt
Abs254 nm
80S
40S
60S
Polysomes
10%
Sucrose gradient
50%
C
Repeat 2
Repeat 3
wt+aal1-pOE
wt+evc
aal1Δ +evc
aal1Δ+aal1-pOE
Abs254 nm
80S
40S
60S
Polysomes
10%
Sucrose Gradient
50%

 © The Author(s)

Figure EV4. *aal1* associates with ribosomes and reduces the cellular ribosome content.

(**A**) Polysome fractionation followed by RT-qPCR shows that *aal1* binds to ribosomes during early stationary phase (Day 0, left graph) and late stationary phase (Day 6, right graph) in both wild-type (green) and *aal1-pOE* (red) cells. (**B**) Independent biological repeats of polysome profiling as in Fig. 4A,B for proliferating cells (top), early stationary-phase cells (middle) and late stationary-phase cells (bottom) for the four strains indicated. The two profiles are aligned at the lowest points of the monosome peaks, corresponding to the baseline. (**C**) Two independent biological repeats of experiment shown in Fig. 4C.

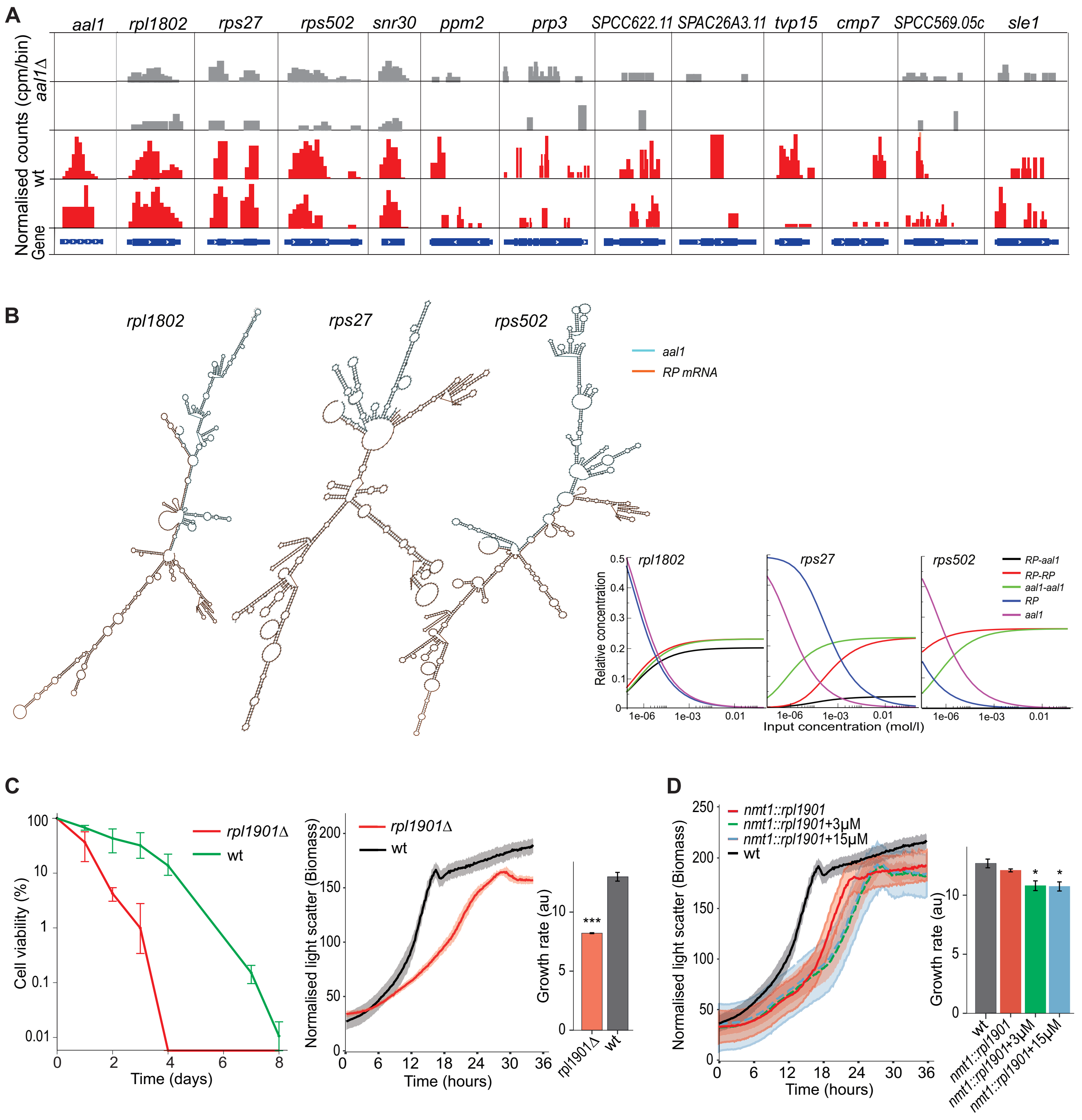

Figure EV5. Analyses of RNAs binding to *aal1*.

(**A**) IGV tracks from strand-specific ChIRP-seq reads in counts per million (cpm) per 50 bp bins (deepTools) (Ramirez et al, 2016) across *aal1* (control) and prospective target RNAs as indicated on top. Top *aal1*-bound RNAs were determined with edgeR (Robinson et al, 2010) (Dataset EV4) from two replicates each of *aal1Δ*, wild type (wt) and *aal1-pOE* cells, and the data were verified in IGV (Thorvaldsdottir et al, 2013) (details in Methods). The prospective *aal1*-bound RNAs in the ChIRP-seq data include three additional mRNAs encoding ribosomal proteins and one small nucleolar RNA (*snr30*). (**B**) In silico predictions of interaction between *aal1* and *rpl1802, rps27, and rps502* using the ViennaRNA package (Lorenz et al, 2011) with RNAcofold (Lorenz et al, 2016). Left: Predicted *aal1-RP* (ribosomal protein) mRNA heterodimers with interaction sites and potential RNA secondary structures. Right: Concentration dependency plots of dimerization showing the computed homo- and hetero-dimerizations of RNAs for concentration relative to each other (y-axis) and different input concentrations (x-axis), with predicted equilibrium concentrations for the monomers, homodimers, and heterodimers as indicated. (**C**) Left graph: Chronological lifespan assays for *rpl1901Δ* and wild-type cells, performed in rich medium. Right graphs: Growth assays of *rpl1901Δ* and wild-type cells and quantitation of growth rate for these assays. Experimental setup and analysis as in Fig. 1D. Statistical significance was determined with one-way ANOVA followed by Dunnett's test (Hothorn et al, 2008), with $p < 0.0001$ relative to wt. (**D**) Growth assays of *nmt1:rpl1901* and wild-type cells with the addition of different doses of thiamine as indicated and quantitation of growth rate for these assays. Experimental setup and analysis as in Fig. 1D. Statistical significance was determined with one-way ANOVA followed by Dunnett's test (Hothorn et al, 2008), with $p < 0.006$ relative to wt.

 © The Author(s)

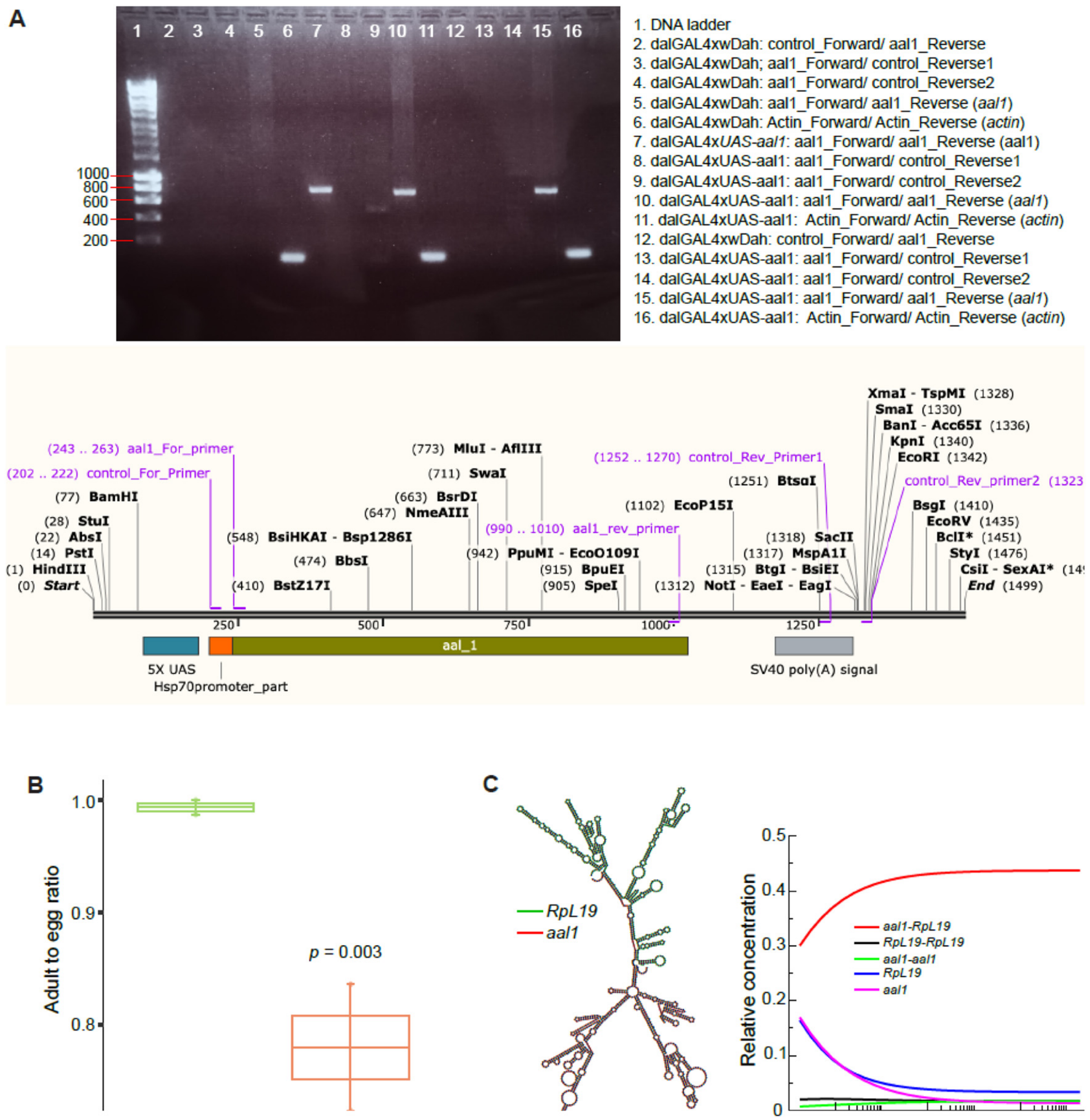

Figure EV6. Supporting analyses for experiment expressing *aal1* in flies.

(**A**) Top: Confirmation of *aal1* expression in the inducible *UAS-aal1* strain flies. The expression of an RNA of expected size (783 nt) was confirmed in females where *UAS-aal1* was driven by the ubiquitous, constitutive GAL4 driver (*daughterlessGAL4/dalGAL4*). Random primed cDNA was used as template. Primer positions as follows (see scheme). Forward control: resides in Hsp70 promoter; Reverse_control1: in SV40 polyA signal; Reverse_control2: in downstream sequence of SV40 polyA signal; aal1_Forward & aal1_Reverse: in 5′ and 3′ ends of *aal1* transcript, respectively; Actin_Forward/Actin_Reverse: housekeeping Actin gene. wDah control crossed to dalGAL4 was used as a negative control strain (Lanes 2–6 and 11). Three *UAS-aal1* replicates were tested (lanes 7, 8–11, 13–16). Primer sequences are provided in Appendix Table S1. Bottom: Scheme of *UAS-aal1* construct showing the positions of the primers used (purple), visualized with SnapGene Viewer 5.3.2. (**B**) Ubiquitous expression of *aal1* in flies with a *dalGAL4* promoter throughout development significantly reduces the number of flies that reach adulthood (details in Methods: Lethality test in flies). Statistical significance determined with two-sample t-test. (**C**) In silico prediction of interaction between *S. pombe aal1* and *Drosophila RpL19* using the ViennaRNA package (Lorenz et al, 2011) with RNAcofold (Lorenz et al, 2016). Left: Predicted *aal1-RpL19* heterodimer structure showing the interaction sites along with potential RNA secondary structures. Right: Concentration dependency plot of dimerization showing the computed homo- and hetero-dimerizations of RNAs for concentration relative to each other (y-axis) and different input concentrations (x-axis), with predicted equilibrium concentrations for the two monomers, *aal1* and *RpL19*, the two homodimers, *aal1-aal1* and *RpL19-RpL19*, and the *aal1-RpL19* heterodimer.

© The Author(s)
